# Deep learning of chest X-rays can predict mechanical ventilation outcome in ICU-admitted COVID-19 patients

**DOI:** 10.1038/s41598-022-10136-9

**Published:** 2022-04-13

**Authors:** Daniel Gourdeau, Olivier Potvin, Jason Henry Biem, Florence Cloutier, Lyna Abrougui, Patrick Archambault, Carl Chartrand-Lefebvre, Louis Dieumegarde, Christian Gagné, Louis Gagnon, Raphaelle Giguère, Alexandre Hains, Huy Le, Simon Lemieux, Marie-Hélène Lévesque, Simon Nepveu, Lorne Rosenbloom, An Tang, Issac Yang, Nathalie Duchesne, Simon Duchesne

**Affiliations:** 1 CERVO Brain Research Center, Québec, Québec, Canada; 2 Physics Department, Université Laval, Québec, Québec, Canada; 3 Department of Radiology and Nuclear Medicine, Université Laval, Québec, Québec, Canada; 4 Department of Family and Emergency Medicine, Université Laval, Québec, Québec, Canada; 5 Centre de recherche intégrée pour un système apprenant en santé et services sociaux, Lévis, Québec, Canada; 6 Centre de recherche sur les soins et les services de première ligne de l’Université Laval, Québec, Québec, Canada; 7 Centre hospitalier de l’Université de Montréal, Montréal, Canada; 8 Electrical and Computer Engineering Department, Université Laval, Québec, Canada; 9 Jewish General Hospital, Montréal, Canada; 10 Department of Diagnostic Radiology, McGill University, Montréal, Canada; 11 Institut universitaire de cardiologie et de pneumologie de Québec, Québec, Canada; 12 Public Health Directory, Centre intégré universitaire santé et services sociaux de la Capitale Nationale, Québec, Québec, Canada

**Keywords:** Radiography, Computer science

## Abstract

The COVID-19 pandemic repeatedly overwhelms healthcare systems capacity and forced the development and implementation of triage guidelines in ICU for scarce resources (e.g. mechanical ventilation). These guidelines were often based on known risk factors for COVID-19. It is proposed that image data, specifically bedside computed X-ray (CXR), provide additional predictive information on mortality following mechanical ventilation that can be incorporated in the guidelines. Deep transfer learning was used to extract convolutional features from a systematically collected, multi-institutional dataset of COVID-19 ICU patients. A model predicting outcome of mechanical ventilation (remission or mortality) was trained on the extracted features and compared to a model based on known, aggregated risk factors. The model reached a 0.702 area under the curve (95% CI 0.707-0.694) at predicting mechanical ventilation outcome from pre-intubation CXRs, higher than the risk factor model. Combining imaging data and risk factors increased model performance to 0.743 AUC (95% CI 0.746-0.732). Additionally, a post-hoc analysis showed an increase performance on high-quality than low-quality CXRs, suggesting that using only high-quality images would result in an even stronger model.

## Introduction

When severe COVID-19 cases require admission to intensive care units (ICU) for respiratory distress and hypoxaemia, endotracheal intubation and mechanical ventilation remain recommended^1^. Throughout the pandemic, the number of cases has at times outstripped the capacity of local health care systems, a situation still relevant in many countries as new variants emerge and vaccination rates and/or efficacy differ. In these instances, ICUs face scenarios where there are insufficient resources for the number of critically sick patients-COVID-19 or otherwise - requiring ventilation, thus requiring a delicate but necessary adjudication of these resources. In that situation, guidelines (for example, see Ehni et al^2^) issued by health authorities have usually in common the principle to withhold care from those with the highest mortality probability given an intervention. This requires however an objective assessment of the latter, based on formalized clinical decision scores.

While such work exists for other conditions, the prediction of the eventual outcome of mechanically ventilated COVID-19 patients remains an open question which our work has attempted to address. As we witnessed in the current literature, and as reported in an on-going review by Wynants et al. (twice updated^3^) of more than 50 prognostic models developed in patients with a diagnosis of COVID-19, four risk assessment models using a combination of clinical data and laboratory markers have been proposed to predict possible outcomes from the time of admission, including the necessity of mechanical ventilation. However, none have addressed the outcome of mechanically ventilated patients per se.

Further, in only one of these models did authors make use of radiological markers (specifically, computed tomography)^4^. Radiology, unlike laboratory tests, can provide an anatomical representation of the distribution and severity of pulmonary disease. As reported, the primary findings for COVID-19 on computed tomography in adults comprise a) bilateral, subpleural, and peripheral ground-glass opacities; (b) crazy paving appearance (ground-glass opacities and inter-/intra-lobular septal thickening); (c) air space consolidation; (d) bronchovascular thickening; and traction bronchiectasis^5–7^. However, the standard of care in the ICU, especially in situations of overcapacity, will be chest x-rays (CXR) performed at the patient’s bedside. There are fewer reports on COVID-19 appearance on CXR, and fewer still focusing specifically on anterior-posterior (A-P CXR) images (of the type taken at the bedside) however, reports indicate patchy or diffuse asymmetric airspace opacities, similar to other causes of coronavirus pneumonias^8^. The main finding over time on CXR (i.e. multi-day imaging) was consolidation^9^.

New research yielded COVID-19 specific clinical scores based on CXR such as the BRIXIA score^10^ that has shown moderately high predictive power in assessing mortality but not specifically after ventilation^11^. Regardless, these reports indicate that radiology, including CXR, provides additional predictive information to standard clinical signs and laboratory findings.

As stated, none of these approaches seems to have been developed specifically to predict mortality as an outcome in patients about to be ventilated, which would be informative for resource adjudication in a triage situation. An automated and reproducible analysis based on machine learning would be desirable to support overworked medical professionals in these situations. Machine learning has been successfully applied to large-scale CXR analysis, notably to predict radiological findings (e.g. ChexNet^12^, MIMIC-CXR^13^) in the pre-pandemic, hospital-admitted patient population. The feasibility of machine learning-powered triage applications based on CXR appearance has also been established^14^.

### Related works

Several studies have highlighted the large potential for bias in most COVID-19 prognosis applications^3,15^. Convenience and open-source datasets are especially prone to being biased, notably due to selection bias, unclear COVID-19 status, combining several datasets with different definitions/outcomes or training and testing on populations with different demographics/risk factors. As such, designing a successful COVID-19 model requires a systematically collected dataset with a well-defined population to limit the risks of bias. Such a dataset, however, would not have the thousands of patients that are typically used to create deep learning models. Using transfer learning, the learned CXR-specific domain knowledge from large scale datasets could be leveraged and applied to smaller COVID-19 CXR datasets. A previous work^16^ demonstrated the feasibility of developing COVID-19 models on small datasets using transfer learning for COVID-19 radiological severity assessment and progression. Parts of the literature also used transfer learning in assessing radiological severity of the disease^16–18^ and predicting the potential for adverse events^19^ for hospital-admitted patients; none have tackled outcome once on ventilation. Furthermore, most prognosis models are either based on imaging data or demographics/risk factors alone. Combining those sources could improve model performance and help determine how much imaging adds to the predictive power of known risk factors. Some publications have studied triage applications for COVID-19 in the optics of minimizing the time until a proper diagnosis is obtained^20^, but the problem of resource allocation in case of an overflow of patient remains.

### Study contributions

The objective of this study was to determine whether a machine learning analysis of CXR taken at the patient’s bedside would be predictive of the outcome of invasive ventilation in the context of COVID-19; and if this information would add to or confirm the predictive ability of other known risk factors of mortality for COVID-19. We test this hypothesis on a systematically collected, multi-center patient cohort using CXR examinations that are already acquired as part of the standard of care and compare the predictions with the demographics and risk factors hazard ratios extracted from large scale studies. Our results suggest that such an automated system designed to identify patients that are unlikely to survive even when treated with mechanical ventilation could benefit triage decisions at the ICU. Furthermore, we demonstrate the performance drop in the model’s predictions when applied to low quality images.

## Materials and method

This is a retrospective study of clinical cases approved by the ethics committee of all participating hospitals (CIUSSS-Capitale Nationale certificate #MP-13-2020-2025). Informed consent was waived due to the retrospective nature of the study. The experimental design was elaborated prior to data collection. This study is conducted and reported based on the STARD criteria^21^. The collected data was first presented in another publication. where it was used for severity prediction^16^.

### Participants

We collected data on all adult patients admitted to ICU for confirmed COVID-19 which were intubated and received invasive ventilation as part of treatment procedure at five Quebec hospitals (Centre hospitalier universitaire de Québec; Hotel-Dieu de Lévis; Institut universitaire de cardiologie et pneumologie de Québec; Jewish General Hospital, Centre hospitalier universitaire de Montréal) in the recruitment period. The canonical index test for COVID-19 presence was the polymerase chain reaction test.

### Outcome

The primary outcome measure of the study was defined as the treatment outcome of either full recovery (i.e. hospital discharge, not simply ICU discharge) or mortality. Images assessors were blinded to outcome. Model creators were not involved in data gathering but were not blind to outcome, since they created the training/testing groups.

### Mortality score

Clinical variables and risk factors play a large role in determining disease outcome, with the elderly being particularly vulnerable. Large-scale studies in the UK have reported hazard ratios associated with a large range of risk factors such as cardiovascular disease, cancer, age or sex^22^. The importance of those risk factors cannot be deduced from our dataset; we used therefore hazard ratios from Williamson et al.^22^ to create a risk factor aggregate named the non-imaging mortality score (MS). The mortality score was defined as the sum of the logarithm of the published hazard ratios for all the available risk factors:

1 $$\begin{aligned} MS = \sum {log(HR)} \end{aligned}$$

**Figure 1:**
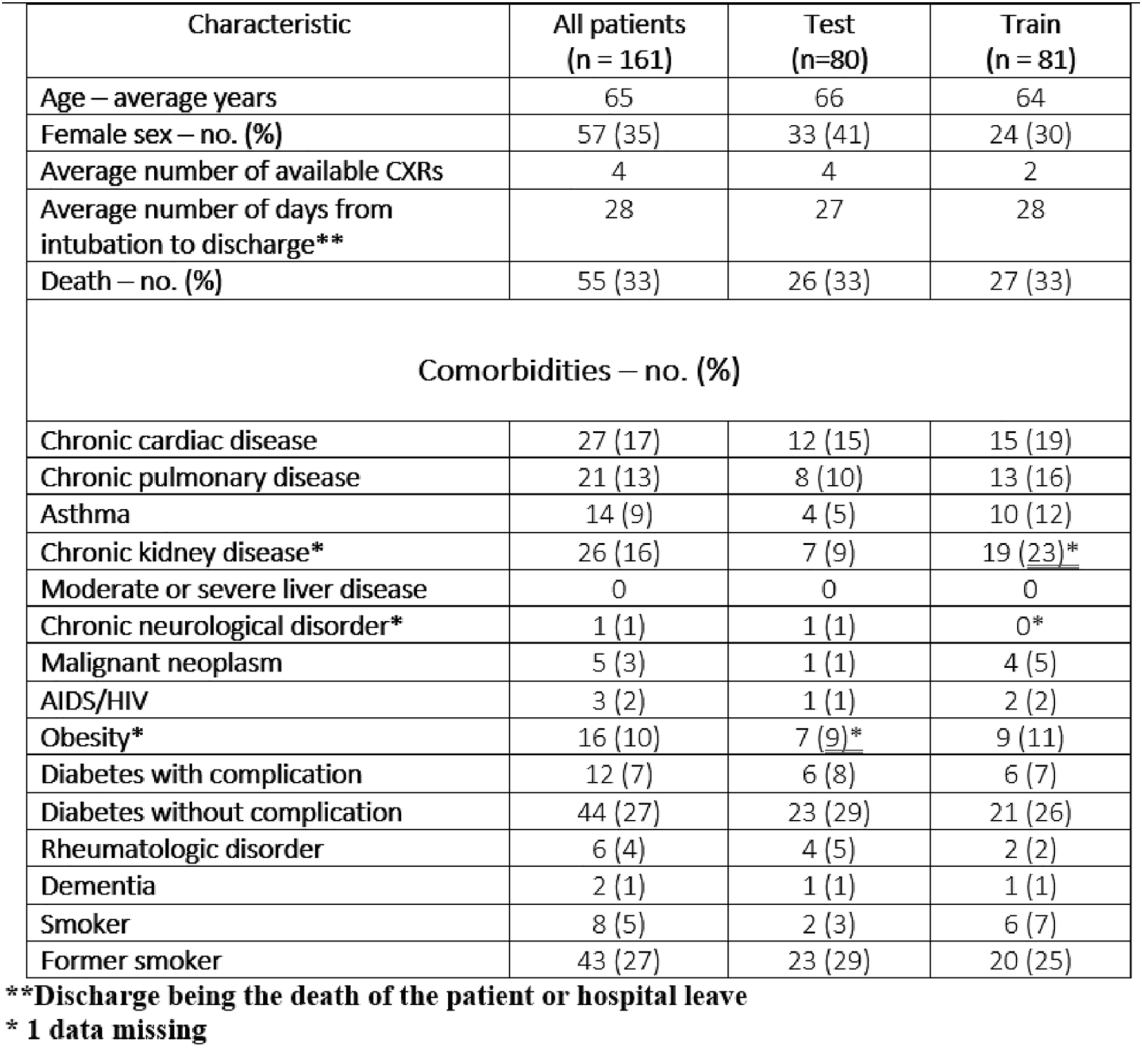
Training and testing set statistics. The * denotes a comorbidity where information about the comorbidity was not available for a single patient.

The clinical variables entering in the MS were collected by co-authors from available patient hospital records using a centralized REDCap interface and are listed in Figure 1.

### Modeling

A supervised deep learning approach, relying on transfer learning, was used to analyse CXR with respect to patients’ outcome. We will first define the various datasets on which the system was trained, validated and tested, before describing the algorithms employed. The model development process is similar to that of a previous publication^16^.

#### Transfer learning dataset

The CheXpert dataset^23^, comprised of 224,316 X-ray images collected from 65,240 unique patients at Stanford hospital, was used to train a feature extractor suited for CXRs (https://stanfordmlgroup.github.io/competitions/chexpert/). It includes images taken in various patient positions and orientations, making it suitable for use on general CXRs. The labels are generated by automatic text mining in the radiology reports. The CheXpert dataset includes a curated validation set with 234 images that is used to evaluate the network’s performance. Three board-certified radiologists from the (Stanford) CheXpert team individually assigned radiological findings and diagnoses to each of the studies in this validation set for increased label reliability.

#### COVID-19 dataset

All patients included in our study received periodical CXRs as part of the standard-of-care while intubated. At a minimum, each patient had one CXR done immediately after intubation in order to assess proper positioning. The other CXRs were obtained based on local systematic standard of care or as clinically required. The patients in the COVID-19 dataset were randomly split in two training and testing datasets in a 1:1 proportion using stratified sampling based on the mortality score computed using the clinical variables. The negative outcomes (deaths) were evenly split between training and testing to have a robust estimate of the performances. Figure 1 presents some comparative statistics between the training and testing groups. The training set used all available pre and post-intubation CXRs, while the testing set contained only pre-intubation CXRs (including the CXR taken at intubation). The dataset was manually curated to remove very-low quality images, such as a significantly overexposed CXRs or images where medical apparatus was overshadowing a large portion of the lungs. CXRs that were 0-padded or rotated following extraction from the picture archiving and communication system (PACS) were manually cropped to remove the padding and center the image on the lungs. Every image in the dataset was also graded for quality on a 0 to 7 scale according to the modified European quality assessment criterion presented in^24^ by a radiology resident.

#### Transfer learning model

The CXRs were first pre-processed by histogram equalization followed by resizing to a 320x320 size. To extract meaningful features from the CXRs, we then used the PyTorch library to train a DenseNet121 supervised network on the CheXpert dataset, as it was reported to yield the best results on this dataset^23^. We did not include the labels “No Findings” and “Fracture” in the classification task due to their lack of relevance in this clinical problem. We further removed “Pneumothorax”, as it was reported to have a very low rate of incidence in COVID-19 patients^25^. Image augmentation was performed by randomly cropping the input images to a 300x300 size. We used the Adam optimizer with default parameters and the learning rate was fixed to 1 × 10^−4^. A batch size of 16 was used. We used the U-zeroes policy as in Irvin et al.^23^ to replace uncertain findings with negative findings. The cost function was a binary cross-entropy loss as is appropriate for multi-class classification. We trained for three epochs, saving the model after every epoch and selecting the checkpoint with the lowest loss on the CheXpert validation set.

#### COVID-19 outcome prediction model

The outcome prediction task was then attempted by using the imaging features extracted by the DenseNet model (1024 values corresponding to the average pooling of the last convolutional feature maps). Prior to feature extraction by the network, the COVID-19 CXRs were pre-processed and normalized in a similar fashion as those from the transfer learning CheXpert dataset. Generalization performances were estimated using leave-one-participant-out cross-validation on the training dataset. While the outcome model was only tested on pre-intubation CXRs because it corresponded to the intended use-case, all CXR (pre- and post-intubation) were used for model training because it is expected that the radiological presentation typical of a poor outcome should persist after intubation; this allowed us to increase the training sample. Next, many precautions were taken to limit overfitting in the training dataset. First, data augmentation was performed just like when training the CheXpert network (resizing to 320x320, randomly cropping to 300x300). A total of 100 random crops per CXR were done to obtain an estimate of feature variability. A normalized variance filter was used to select features that were the most stable within the random crops. The features were scaled to a [0,1] range. The remaining features were then ranked according to their correlation with the outcome with the ANOVA F-value, and the best K features selected from all folds. Three types of candidate classifiers (support vector classifier, logistic regression, random forest) were then trained on this feature set and cross-validated. We optimized the amount of variance filtering, the number K of selected features, and the various classifier hyperparameters to maximize the ROC-AUC on the cross-validated samples. The selected combination of model and hyperparameters was then trained on the whole training set and tested once on the testing set.

### Statistical analysis

The performance of the model was then compared to a model using only the mortality score computed using the clinical variables and risk factors. Because it was composed of a single feature, the mortality score model was a simple logistic regression model with no need for hyperparameter optimization. A combined model using both the selected convolutional features and the mortality score was also developed, consisting of the chosen cross-validated model re-trained with the mortality score as an additional feature. Confidence intervals on the testing set were computed by stratified percentile bootstrapping to keep an identical class balance. The Python Sklearn library was used to train the classifiers and do the statistical analysis.

### Quality analysis

It is expected that the model’s performance should vary depending on the CXR’s quality. Intuitively, an overexposed CXR does not contain as much information about the pathology as a well-calibrated one. To investigate the importance of image quality, the testing set was partitioned post-hoc according to the total image quality score to make two groups as balanced as possible.

## Results

### Participants and data

**Figure 2:**
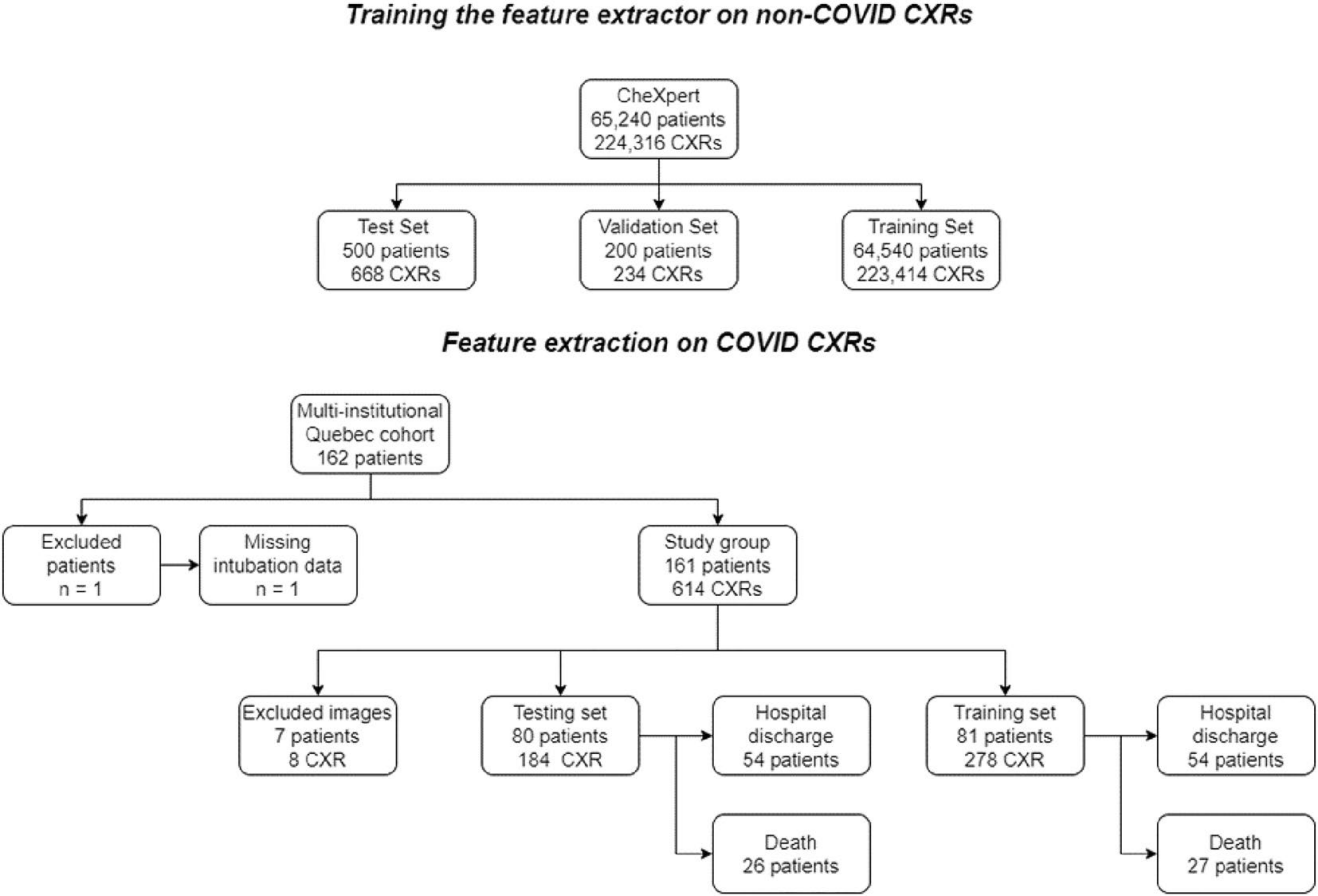
Study flowchart. The CheXpert training set is used to pre-train a general CXR feature extractor that is subsequently used on COVID patients. The CheXpert test set is not publicly available, and results were reported on the validation set.

**Figure 3:**
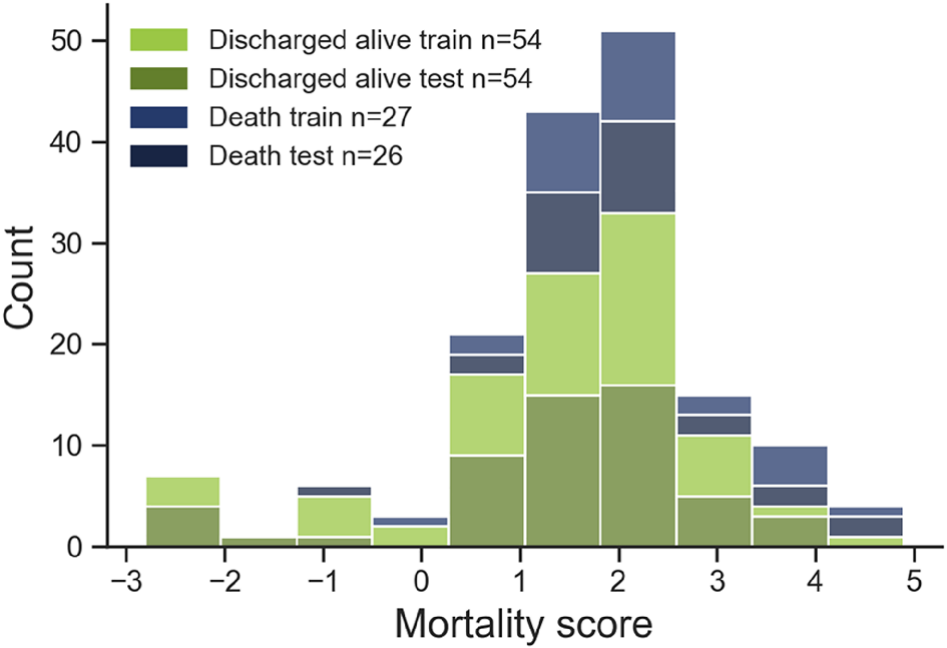
Distribution of the mortality score in the train and test split of the dataset. The split is done using stratified sampling (on the basis of the mortality score) to limit small-sample variance. Deaths and survivals were evenly split between training and testing.

We collected data on 162 adult participants (M/F: 105/57, mean age 65) admitted to ICU for confirmed COVID-19 and that received invasive ventilation between March 2020 and February 2021. One participant was excluded due to an unknown intubation date, as the intubation was performed at an outside institution. The remaining 161 patients (Figure 2) received periodical CXRs while intubated for a total of 614 CXRs. Eight images were removed due to poor quality. The training set contained 278 CXRs. In the testing set, 184 images are pre-intubation CXRs. The group of high-quality images contained 73 images with a score higher than 5, while the low-quality group contained 121 images with a score of 5 or less. 53 participants died while 108 were discharged alive. The mean days between intubation and discharge or death was 28 days. Figure 3 presents the histogram of the mortality scores in the training/testing folds.

### Analysis

#### Results on the validation set

**Figure 4:**
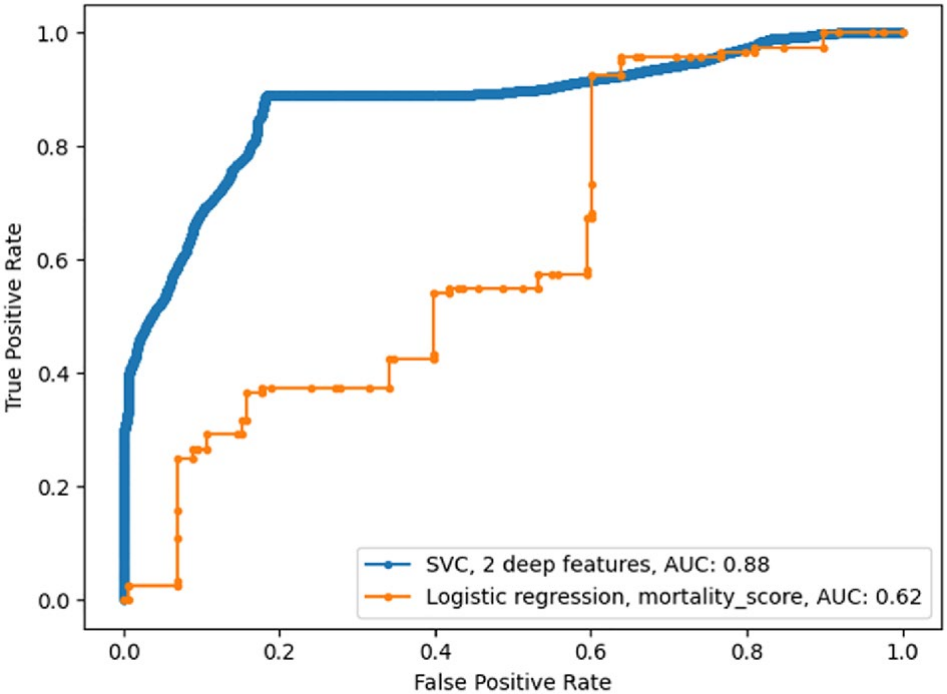
Cross-validated performances of the developed models. The best-performing classifier for the imaging features was a support vector classifier using 2 features with a linear kernel, achieving a 0.88 AUC. The mortality score model obtains a 0.62 AUC with a logistic regression model. There are 152 positive samples and 126 negative samples in the validation set.

The selected model using imaging features was support vector classifier using the best two convolutional features, a linear kernel and a C of 6E-4. The model used a balanced learning strategy to assign a higher cost to misclassified examples in the minority class. Figure 4 presents the ROC curves for the cross-validated models. The model using the imaging features had a higher ROC-AUC (0.88) than the mortality score model (0.62). The ROC curve for the imaging features model is smoother because of data augmentation.

**Figure 5:**
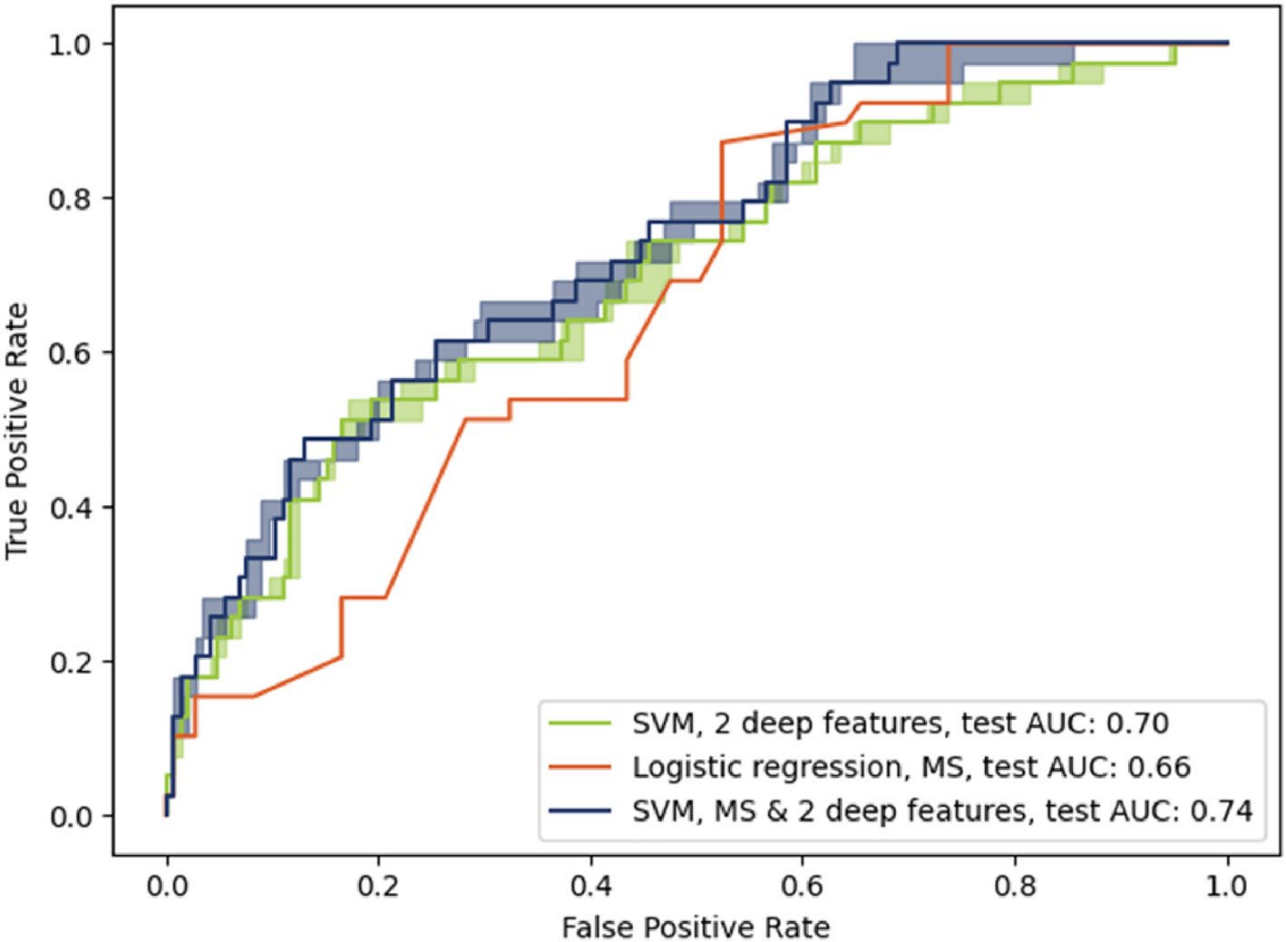
Testing performances of the developed models. The model using the deep imaging features performed worse on the testing set than on the validation set (0.70 compared to 0.88), pointing to some overfitting. In contrast, the mortality score model similarly (0.66 compared to 0.62). Combining the imaging features with the mortality score yielded a slightly higher AUC of 0.74, using the same SVM classifier. There are 145 positive samples (survival) and 39 negative samples (Deaths) in the testing set.

#### Results on the testing set

**Figure 6:**
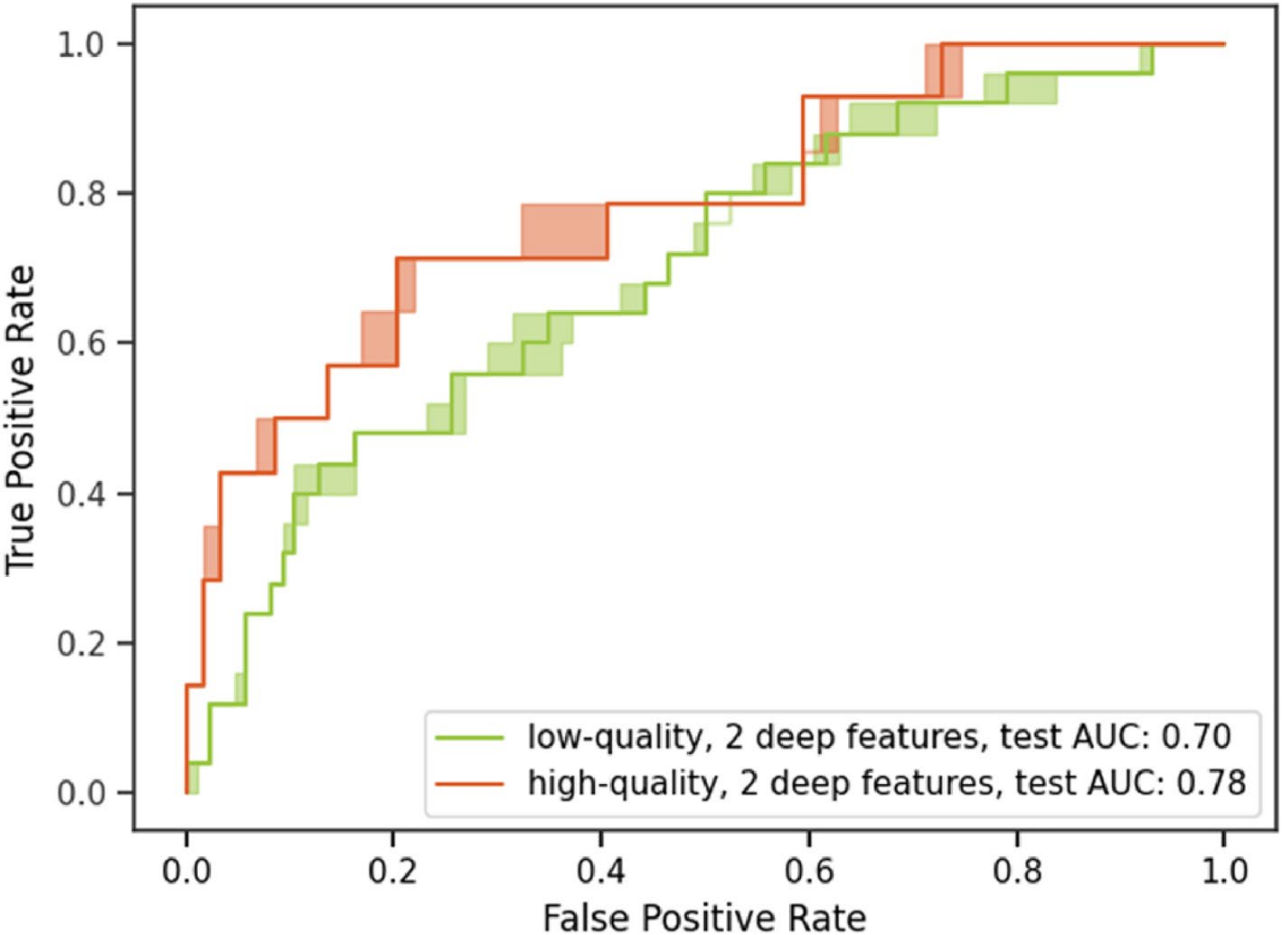
Testing performances of the imaging features on low and high-quality images. The model’s prediction on high-quality images (Score of 6-7) have a slightly higher AUC than on lower quality images.

Figure 5 presents the ROC curve for the models when applied on the testing set. In this case, the ROC was constructed using the average prediction for the 100 random crops per image. A decreased AUC (0.702, 95% CI 0.707-0.694) was obtained for the imaging features model on the testing set, compared to the cross-validated results (0.88), while a similar AUC was obtained for the mortality score (0.663). The model using the combined features scored highest (0.743, 95% CI 0.746-0.732). The comparable AUC in the training and testing sets for the mortality score model can be attributed to the dataset splitting that was designed to ensure a similar distribution in mortality scores for both folds. The effect of the image quality on the AUC of predictions is presented in Figure 6. Predictions on high-quality CXRs (score of 6-7) have a higher AUC of 0.776 (95% CI 0.783-0.748) compared to the lower-quality CXRs (score of< 6) that have an AUC of 0.701 (95% CI 0.707-0.687). Binary classification measures for the chosen models are compiled in Table 1. The operating point for each model is set to obtain a 50% sensitivity. The combined model using the mortality score shows a marginal improvement. The imaging features and the mortality score predict the same outcome for 126 out of 184 CXRs (69%) in the testing set, with an 81% accuracy on that subset of patients. As such, the predictions are not highly correlated, indicative of their providing different, complementary information.

**Figure 7:**
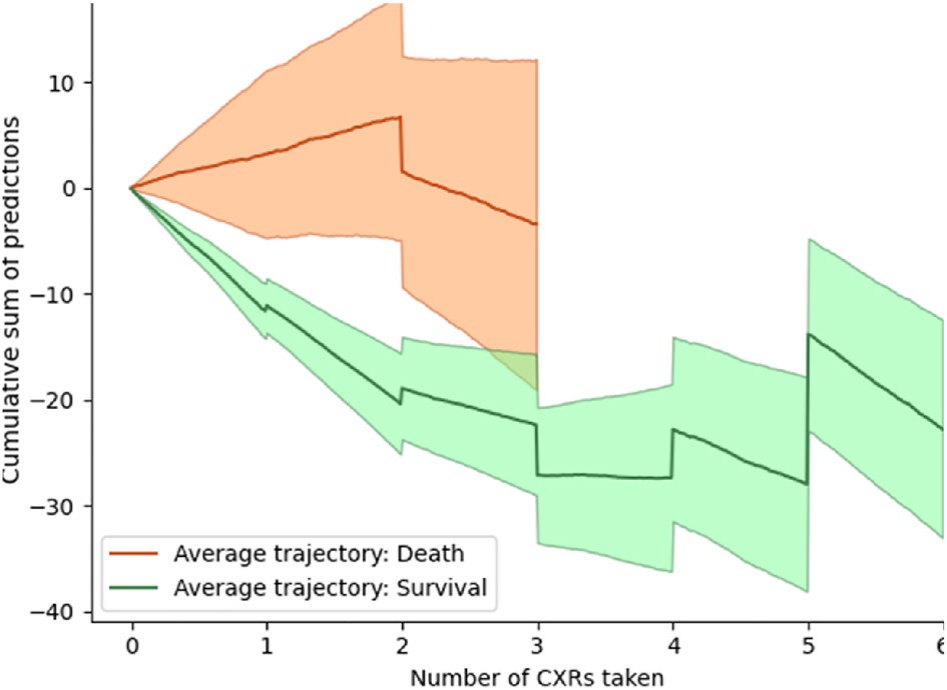
Average patient trajectory in the testing set by outcome. The cumulative sum of the imaging model predictions over repeated CXRs enabled the tracking over time, post-intubation, of the potential recovery of patients. The shaded area represents a standard deviation, with discontinuities in the curve when the number of patients change. The curves are stopped when only three patients are left in each outcome group.

An advantage of CXR imaging over mortality score determination from risk factors is the potential for repeated measurements and evolution tracking. Figure 7 presents the average trajectory for the cumulative sum of predictions over repeated CXRs for both categories of patients. The potential to keep tracking a patient’s state over multiple CXRs following intubation is promising.

**Table 1:** Binary classification performances of the proposed models on the testing set.

Model	Accuracy (%)	Sensitivity (%)	Specificity (%)	PPV (%)	NPV (%)
Imaging features	75.0	51.3	81.4	43.6	86.1
Mortality Score	67.4	51.3	71.7	32.8	85.6
Combined	75.5	48.7	82.8	43.3	85.7

## Discussion

We propose an algorithm to assist decision making at the ICU for immediate ventilation and to assist allocation of resources in case of scarcity, or, for other healthcare systems (e.g. lack of resources) guide decisions on case transfer. Designed to work with a small amount of data, the proposed model is a linear support vector classifier using two imaging features extracted using a deep neural network pre-trained on a large corpus of non COVID-19 CXRs. Despite some overfitting on the validation set, the model reaches better results than the use of aggregated risk factors such as age, sex and comorbidities (0.707 AUC compared to 0.663). Imaging features provided complementary information to the non-imaging risk factors, with the combined model reaching a 0.74 AUC.

### Generalizability

The dataset is very well-suited to the prediction task, as it was collected systematically and tested in a multi-centric, multi-vendor situation. Contrary to the commonly used open-source, convenience COVID-19 datasets like the ‘COVID-19 image data collection’^26^, ours was collected in a systematic fashion with the proposed application in mind. Therefore, the model should be directly applicable to the population under study. As pointed out in a review of COVID-19 AI applications, most prediction models are at high risk of overfitting or bias due to the low number of negative outcomes^4^. We addressed this issue by pre-training the model on non-COVID CXR and not retraining the DenseNet. Furthermore, the use of data augmentation and feature selection yielded a small model using only two features and tight confidence intervals (+/- 0.01 AUC). Thus, our model should be at a low risk of bias considering the commonly used criteria of 20 events per variable^15^, even if the total number of examinations in the training set is relatively low (278). It is important to note that these confidence intervals do not account for the intrinsic noise in the composition of the testing set. As such, we can assume the model’s robustness to variations in the training set, but the model’s performance in prospective use could be out of the computed confidence intervals. Care must be taken in interpreting the results of the model. It aims to answer a specific, well-defined question, namely: would a given ICU-admitted COVID-19 patient survive intubation? It has been validated and tested on a specific population of patients and might not perform as well outside of it. It should not be used to predict general COVID-19 mortality, and its results might be less reliable in edge cases such as pediatric patients who are outside the age distribution of the training set and whose CXR appearance might significantly differ.

### Comparisons

The results are not directly comparable with other COVID-19 prognosis works because of the different sub-population that is under study and different sample sizes. Sriram et al.^19^ reported AUCs for mortality prediction around 0.81 for 4-day mortality using sequences of images (mortality prediction AUC for a single image was not reported). The lower AUC of 0.743 than on the general hospital-admitted population is not surprising because the average severity in this dataset should be higher and the different outcomes groups have a radiological presentation that is closer in appearance. These results show that a data-driven COVID-19 prognosis model can be developed with a smaller amount of data than has been previously done while maintaining good performance. For instance, the chosen SVM is only trained on 278 CXRs, compared to the 17,915 in^19^ or 5617 in^27^. On the other hand, the model’s applicability is narrower and restricted to pre-intubation ICU patients. The slight improvement in model quality when incorporating age into the model is comparable to what was reported by^10^. The comparatively high AUCs (0.85 for BRIXIA score and risk factors, compared to 0.74 for our method) reported in^10^ are not obtained in the same conditions and not for the same population. For each patient, the radiograph with the highest BRIXIA score was selected, which is not a method applicable in prospective studies as it cannot be known when the radiological severity has peaked. Comparatively, our method has a clear and clinically relevant point of entry, with the model output giving useful information for a clinical decision to be made at that time. Future works could approach the auxiliary task of predicting the length of stay at the ICU, which combined with the chances of survival would also be useful information for a triage committee.

### Limitations

The main limitation of this work arises from the small number of patients that limit the strength of our results. It is likely that the dataset does not cover all possible severe COVID-19 CXR presentations. Another potential limitation to the usefulness of the model is the evolution of the standard-of-care for COVID-19 patients experiencing respiratory distress. As the best practices changed significantly since the onset of the pandemic, we can expect the survival rate to be higher if this model was used in a prospective study. A significant limitation to the prediction accuracy of the proposed model is the quality of the radiographs. The quality of portable bedside CXRs depends on multiple factors, such as patient body habitus, patient positioning, technologist operator experience and the quality of the portable x-ray machine. While newer technologies used in portable x-ray machines have improved image quality, the other variables may affect overall diagnostic quality. Some CXRs were rejected for overexposure, but there was still a high variability in CXR quality and contrast in the dataset. Our quality evaluation of the CXRs showed that the model tends to perform better on higher quality images with a gain of 0.08 AUC on this subpopulation. It is expected that training using high-quality CXRs with a consistent contrast should result in a higher-performing model, although it would probably generalize poorly on low-quality CXRs. Nonetheless, in these challenging conditions, we can still establish that CXRs contain useful information and features that can be used for the prognosis of COVID-19 patients experiencing respiratory distress in the ICU.

## Conclusion

In conclusion, this work demonstrates the feasibility of using deep transfer learning in a low sample regime for the development of COVID-19 mortality prediction. This represents a potentially useful tool to provide an objective estimate of mortality probability to inform proper resource allocation in a hospital overflow situation. We used multi-centric data collected in a systematic fashion to accurately describe a specific patient population. A simple model using only two features and having tight confidence intervals (AUC: 0.702, 95% CI 0.707-0.694) is described and can be combined with known COVID-19 clinical risk factors for increased performances (AUC: 0.743, 95% CI 0.746-0.732).
